# Extending the Technology Acceptance Model: Digital Literacy Adoption, Academic Performance, and Professional Preparedness Among Nursing and Midwifery Students in Ghana

**DOI:** 10.1155/nrp/4846118

**Published:** 2026-11-14

**Authors:** Alhassan Shaibu, Iddi Anass

**Affiliations:** ^1^ Department of Business Analytics and MIS University for Development Studies Tamale Ghana uds.edu.gh; ^2^ Department of Finance University for Development Studies Tamale Ghana uds.edu.gh

**Keywords:** academic performance, digital literacy, Ghana, midwifery education, nursing education, PLS-SEM, professional preparedness, TAM, technology acceptance model

## Abstract

**Purpose:**

This study examines the associations between digital literacy adoption, academic performance, and professional preparedness among nursing and midwifery students at the Nursing and Midwifery Training College, Tamale, Ghana. Grounded in the Technology Acceptance Model (TAM), the study tests perceived ease of use and perceived usefulness as antecedents of digital literacy adoption and traces the hypothesized pathway from adoption through academic performance to professional preparedness.

**Design/Methodology/Approach:**

A quantitative, cross‐sectional survey design was adopted. Primary data were collected from 174 nursing and midwifery students using a structured questionnaire in which all constructs, including academic performance and professional preparedness, were measured through self‐report. Data were analyzed using Partial Least Squares Structural Equation Modeling (PLS‐SEM) in SmartPLS 4, following the two‐step assessment protocol of Hair et al. (2019). Direct paths were tested by bootstrapping with 5000 subsamples. Specific indirect effects were taken directly from the bootstrap with 95% percentile confidence intervals, effect sizes were obtained from the native SmartPLS *f* ^2^ routine, predictive power was assessed with PLSpredict, and common method variance was screened using the full collinearity procedure of Kock (2015). A sensitivity analysis excluding the 50 pilot respondents is also reported.

**Findings:**

All seven hypothesized associations reached statistical significance in the full sample (*n* = 174). Perceived ease of use was positively associated with academic performance (H1: *β* = 0.142, *p* = 0.013), perceived usefulness (H2: *β* = 0.779, *p* < 0.001), and adoption (H4: *β* = 0.183, *p* = 0.009). Perceived usefulness was positively associated with academic performance (H3: *β* = 0.438, *p* < 0.001) and adoption (H5: *β* = 0.678, *p* < 0.001). Adoption was positively associated with academic performance (H6: *β* = 0.348, *p* < 0.001), which in turn was strongly associated with professional preparedness (H7: *β* = 0.753, *p* < 0.001). When the 50 pilot respondents were excluded (*n* = 124), the direct association between perceived ease of use and academic performance (H1) was no longer significant; the other six associations remained significant. The model explains 75.1% of the variance in self‐rated academic performance and 56.7% in professional preparedness. Because all constructs were measured by self‐report at a single time point, and three constructs exceeded the full collinearity criterion for common method bias, these estimates may be inflated by common method variance, and the cross‐sectional design does not permit causal inference.

**Originality/Value:**

The study extends TAM to nursing education in a rural, resource‐constrained Ghanaian setting, a context largely absent from the digital literacy literature. It links TAM antecedents to nursing‐specific outcomes and offers guidance for educators, curriculum developers, and policymakers working to build digital competency in healthcare education.

## 1. Introduction

The rapid expansion of digital technologies in the 21st century has made digital literacy an indispensable skill set across all levels of education. Digital literacy goes beyond technical proficiency with devices; it covers the skills required to access, evaluate, produce, and communicate information using digital technology [1]. In higher education institutions, digital literacy is increasingly linked to academic performance, pedagogical transformation, and graduates’ preparedness for professional practice [2, 3].

In the Ghanaian context, the government’s efforts to integrate Information and Communication Technology (ICT) into education are reflected in various national policies, including the Ghana ICT in Education Policy [4]. Despite these efforts, significant inequalities persist, particularly between urban and rural educational institutions [5]. Nursing education sits at the intersection of these challenges: nurses must simultaneously master clinical skills and navigate an increasingly technology‐driven healthcare environment that relies on electronic health records (EHRs), telemedicine, and digital clinical decision support systems [6, 7].

The Nursing and Midwifery Training College, Tamale, is a public preservice nursing and midwifery institution in the Northern Region of Ghana. Established in 1974, it trains both nurses and midwives and offers Diploma in Registered General Nursing, Diploma in Midwifery, and Post‐NAC/NAP Midwifery program. As a public college serving a largely rural catchment, it operates with limited computing infrastructure and unreliable internet connectivity. Like many rural training institutions in northern Ghana, the college faces the dual task of equipping students with clinical competencies and the digital skills required for modern healthcare delivery. While studies have explored digital literacy in higher education broadly [8–10], empirical evidence on its specific effects within rural nursing education contexts remains scarce. In particular, the mechanisms through which digital literacy adoption relates to academic success and professional readiness, and the role of perceived ease of use and perceived usefulness as determinants of adoption, have not been empirically examined in this setting.

This study addresses this gap by applying the Technology Acceptance Model (TAM) (Davis, 1989) to examine: (1) the correlates of digital literacy adoption among nursing and midwifery students; (2) the relationship between digital literacy adoption and academic performance; and (3) the relationship between academic performance and professional preparedness. Using PLS‐SEM with data from 174 nursing and midwifery students, the study tests a seven‐hypothesis model together with the indirect pathways implied by the model, contributing both a theoretical extension of TAM and practical evidence for digital literacy integration in nursing education in developing‐country contexts.

The remainder of this paper is structured as follows. Section 2 reviews the pertinent literature and develops the research hypotheses. Section 3 describes the research methodology. Section 4 presents and discusses the results. Section 5 concludes with implications, recommendations, and directions for future research.

## 2. Literature Review

### 2.1. Digital Literacy in Higher Education

Digital literacy has emerged as a core competency in 21st‐century education. Ng [1] identifies three dimensions: the technical dimension (proficiency in digital devices and software), the cognitive dimension (critical thinking, problem‐solving, and information literacy), and the social–emotional dimension (ethical behavior, digital citizenship, and online communication). The European Commission’s DigComp framework extends this conceptualization to five areas: information and data literacy, communication and collaboration, digital content creation, safety, and problem‐solving [11].

Digitally literate students tend to achieve stronger academic outcomes by using digital tools for research, collaboration, and learning management [12, 13]. Littlejohn et al. [14] show that digital literacy facilitates learner autonomy, enabling students to independently access, curate, and create content matched to their learning goals. However, a persistent digital divide, worsened by socioeconomic inequalities and infrastructure deficits, limits the development of these competencies in developing countries [15, 16]. In Ghana, this divide is particularly pronounced between urban and rural institutions [5].

### 2.2. Technology Adoption in Educational Institutions

Davis’s [17] TAM proposes that technology adoption behavior is primarily driven by two cognitive beliefs: perceived usefulness, the degree to which a person believes a technology improves performance, and perceived ease of use, the degree to which a technology is believed to be free of effort. TAM has been extensively validated across educational and healthcare contexts [18–20]. Venkatesh et al.’s [21] Unified Theory of Acceptance and Use of Technology (UTAUT) extends TAM by incorporating social influence and facilitating conditions, while Rogers’ [22] Diffusion of Innovations (DOI) theory provides a complementary macro‐level explanation of how innovations spread through social systems.

Institutional factors, including infrastructure, administrative support, and faculty training, are strong determinants of technology adoption in educational settings [23, 24]. Barriers include cost, resistance to change, infrastructure limitations, and inadequate professional development for educators [25]. In resource‐constrained settings like northern Ghana, these barriers are amplified by poor internet connectivity, limited access to devices, and reliance on traditional pedagogical approaches [26].

### 2.3. Digital Literacy in Nursing Education

The integration of digital literacy in nursing education has gained urgency with the digitalization of healthcare. Kuek and Hakkennes [7] show that digitally literate nurses report greater efficiency and job satisfaction in EHR adoption. Simulation‐based learning enabled by digital tools improves nursing students’ clinical decision‐making and professional confidence [27, 28]. Access to digital databases (PubMed, CINAHL, and Cochrane Library) supports evidence‐based practice, a cornerstone of quality patient care [29]. During the COVID‐19 pandemic, telehealth platforms sustained continuity in nursing education [30].

Despite these benefits, challenges persist. In low‐resource settings, inadequate infrastructure constrains digital learning opportunities, and gaps in nursing educators’ own digital literacy limit the effectiveness of training [31]. Resistance to change and fear of technology among both students and educators are further barriers to integration [23, 25].

### 2.4. Theoretical Framework: TAM

TAM was selected as the theoretical lens for this study for three reasons. First, its focus on perceived usefulness and perceived ease of use maps directly onto the study’s research objectives regarding adoption determinants. Second, TAM has been validated extensively in educational and healthcare technology adoption contexts [18–20]. Third, its parsimony of two core constructs with strong predictive validity suits settings where more complex models may encounter measurement challenges.

Within the model tested here, perceived ease of use is hypothesized to relate to perceived usefulness, adoption, and academic performance; perceived usefulness to adoption and academic performance; adoption to academic performance; and academic performance to professional preparedness. Table 1 summarizes reviewed studies and the theories applied.

**TABLE 1:** Summary of reviewed studies and theoretical frameworks applied.

Author(s)	Theory used	Relevant findings
Kuek & Hakkennes [7]	TAM	Digital literacy positively relates to attitude toward EHRs, improving efficiency and job satisfaction
Tubaishat [20]	TAM	Perceived ease of use and usefulness significantly predict nurses’ adoption of EHR systems
Singh & Masango [32]	Digital Literacy Framework	Students perceive digital literacy as important for academic success and access to medical information
Barzekar et al. [27]	Simulation Learning Theory	Simulation‐based learning improves clinical skills, decision‐making, and confidence
Eckhoff et al. [30]	UTAUT	Telehealth integration in nursing education continued learning during COVID‐19, despite access challenges
Terry et al. [33]	Digital Literacy Framework	Digital literacy matters for e‐learning engagement, academic performance, and professional development
Tischendorf et al. [31]	DOI Theory	Main barriers include a lack of infrastructure and inadequate digital skills among educators
Melnyk & Fineout‐Overholt [29]	EBP Framework	Digitally literate nurses apply evidence‐based practices more effectively, improving patient outcomes

*Note:* Source: Authors’ construct (2025).

### 2.5. Hypothesis Development

#### 2.5.1. Perceived Ease of Use and Academic Performance (H1)

In its original formulation, TAM does not posit a direct path from perceived ease of use to performance outcomes; ease of use operates through perceived usefulness and use behavior [17, 34]. This study nevertheless includes a direct PEU ⟶ academic performance path, for two reasons. First, cognitive load theory suggests a mechanism that bypasses usefulness perceptions: when digital tools are easy to navigate, cognitive resources are freed for learning content rather than technology management, which may be associated with stronger academic outcomes independently of whether students judge the tools as useful [35, 36]. Second, empirical work in educational settings has reported direct associations between ease‐of‐use perceptions and student performance in mandatory e‐learning contexts [37], suggesting that the standard mediated‐only structure may understate the role of usability where tool use is not optional, as is increasingly the case in nursing curricula. Including the direct path allows the mediation analysis in Section 4.3.4 to test, rather than assume, whether the PEU‐performance association is fully carried by usefulness and adoption.

 H1: Perceived ease of use of digital literacy tools is positively related to nursing and midwifery students’ academic performance.

#### 2.5.2. Perceived Ease of Use and Perceived Usefulness (H2)

TAM posits that ease of use is a prerequisite for perceptions of usefulness; tools that are difficult to use are unlikely to be perceived as beneficial [34]. For nursing students, ease of navigation and interaction with digital tools translates into perceived academic and clinical utility, creating a positive feedback loop in the adoption process [19].

 H2: Perceived ease of use of digital literacy tools is positively related to nursing and midwifery students’ perceived usefulness of these tools.

#### 2.5.3. Perceived Usefulness and Academic Performance (H3)

Perceived usefulness is among the strongest predictors of technology adoption and sustained use in TAM [17]. When nursing students believe digital tools will improve their academic performance, they are more likely to engage with simulations, online learning platforms, and EHRs, which is associated with improved learning outcomes [2, 35].

 H3: Perceived usefulness of digital literacy tools is positively related to nursing and midwifery students’ academic performance.

#### 2.5.4. Perceived Ease of Use and Adoption of Digital Literacy Tools (H4)

Ease of use is a core determinant of technology adoption in TAM [17]. Students who perceive digital literacy tools as easy to operate are more willing to integrate them into study routines, even among those with lower initial digital proficiency [13, 18].

 H4: Perceived ease of use of digital literacy tools is positively related to nursing and midwifery students’ adoption of these tools.

#### 2.5.5. Perceived Usefulness and Adoption of Digital Literacy Tools (H5)

The perceived advantage of technology for academic performance and clinical readiness is a primary motivator for adoption [17, 38]. When nursing students see clear academic and clinical benefits from digital tools, they are likely to adopt them as core components of their learning practice [19].

 H5: Perceived usefulness of digital literacy tools is positively related to nursing and midwifery students’ adoption of these tools.

#### 2.5.6. Adoption of Digital Literacy Tools and Academic Performance (H6)

The adoption of digital tools gives nursing students access to expanded information resources, collaborative learning platforms, and interactive simulations associated with stronger academic performance [37, 39]. Self‐directed digital learning promotes critical thinking and deepens engagement with nursing curriculum content.

 H6: Adoption of digital literacy tools is positively related to nursing and midwifery students’ academic performance.

#### 2.5.7. Academic Performance and Professional Preparedness (H7)

Academic performance in nursing programs reflects the depth of theoretical knowledge and clinical skill acquisition that are foundational to professional competence [29]. High academic achievement is associated with greater confidence, clinical reasoning ability, and readiness for the demands of healthcare practice [40].

 H7: Nursing and midwifery students’ academic performance is positively related to their professional preparedness.

### 2.6. Conceptual Model

Figure 1 presents the conceptual model derived from the TAM‐grounded hypothesis development. Each of the seven hypotheses (H1 to H7) specifies a direct relationship and is drawn as a solid arrow carrying exactly one label. The model also implies mediated pathways, for example, PEU ⟶ PU ⟶ ADL ⟶ AP and ADL ⟶ AP ⟶ PP. These are not stated as separate hypotheses. They are combinations of the direct paths and are assessed through bootstrapped indirect effects in Section 4.3.4 (Table 2).

**FIGURE 1:**
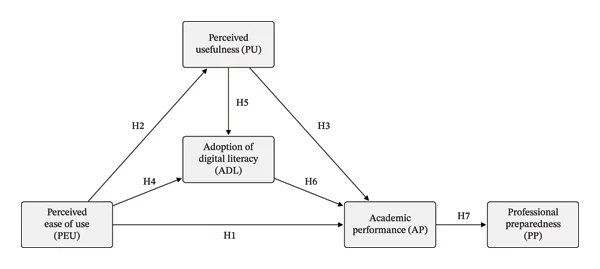
Conceptual model of the study (TAM‐based). Note: H1 = PEU ⟶ AP; H2 = PEU ⟶ PU; H3 = PU ⟶ AP; H4 = PEU ⟶ ADL; H5 = PU ⟶ ADL; H6 = ADL ⟶ AP; and H7 = AP ⟶ PP. PEU = Perceived Ease of Use; PU = Perceived Usefulness; ADL = Adoption of Digital Literacy; AP = Academic Performance; PP = Professional Preparedness. Source: Authors’ construct based on Davis [17].

**TABLE 2:** Specific indirect effects.

Pathway	Indirect effect	SE	*t*	*p*	95% CI
PEU ⟶ PU ⟶ AP	0.341	0.065	5.237	< 0.001	[0.216, 0.472]
PEU ⟶ ADL ⟶ AP	0.064	0.027	2.315	0.021	[0.016, 0.123]
PEU ⟶ PU ⟶ ADL	0.528	0.052	10.251	< 0.001	[0.427, 0.632]
PEU ⟶ PU ⟶ ADL ⟶ AP	0.184	0.045	4.126	< 0.001	[0.102, 0.277]
PU ⟶ ADL ⟶ AP	0.236	0.057	4.165	< 0.001	[0.131, 0.355]
ADL ⟶ AP ⟶ PP	0.262	0.056	4.708	< 0.001	[0.155, 0.375]

*Note:* All quantities are bootstrap estimates from the SmartPLS‐specific indirect effects routine (5000 subsamples, percentile confidence intervals). An interval excluding zero indicates a significant indirect effect. Total effects: PEU ⟶ AP = 0.731 (direct 0.142 plus total indirect 0.588); PU ⟶ AP = 0.674 (direct 0.438 plus indirect 0.236). Source: SmartPLS output (authors’ construction, 2025).

## 3. Research Methodology

### 3.1. Research Paradigm and Design

This study adopts a positivist research paradigm, which holds that knowledge is obtained through empirical observation and scientific inquiry, and that reality is objective and measurable [41]. A quantitative, cross‐sectional survey design was employed, using structured questionnaires [42, 43]. Because all constructs were measured at a single time point, the design tests hypothesized associations derived from TAM; it cannot establish the causal ordering that TAM proposes. This constraint governs the interpretive language used in Sections 4 and 5.

### 3.2. Study Population, Sampling, and Data Collection

The target population comprised students enrolled at the Nursing and Midwifery Training College, Tamale, in the Northern Region of Ghana. The college offers Diploma in Registered General Nursing, Diploma in Midwifery, and Post‐NAC/NAP Midwifery program, and students in all three programs were eligible. Eligible participants were students actively enrolled in any of these programs at the time of data collection; there were no exclusion criteria beyond current enrollment. The questionnaire did not record program of enrollment, so the numbers of nursing and midwifery students among the 174 participants cannot be reported. The sample is therefore described throughout as nursing and midwifery students. The youngest participant was 18 years old, so all participants were adults who consented on their own behalf (Section 3.5).

Sample size adequacy was assessed in two ways. First, an a priori power analysis using G∗Power [44] for a linear multiple regression with three predictors (the maximum number of predictors of any endogenous construct in the model), a medium effect size (*f* ^2^ = 0.15), *α* = 0.05, and power = 0.95 indicated a minimum sample of 119. Second, the sample exceeds the older PLS‐SEM “10‐times rule” [45], which is reported here only for comparability with prior studies, since it is no longer regarded as a sufficient basis for power justification on its own [46]. The 174 usable responses exceed both benchmarks.

Responses were collected in two modes: 150 via Google Forms and 24 via printed questionnaires. The same instrument, item order, and response scale were used in both modes; formal tests of mode equivalence were not conducted, and this is acknowledged as a limitation in Section 5.4. Convenience sampling was adopted given its cost‐effectiveness and suitability for reaching geographically dispersed respondents [47]; the implications of this choice for generalizability are discussed in Section 5.4.

The instrument was piloted with 50 students from the same population to assess item clarity and reliability. Because the pilot produced no changes to item wording, response scale, or item order, pilot responses were retained in the final analytic sample of 174; each pilot participant completed the instrument once. The pilot responses are records R001 to R050 in the dataset. The retention of pilot cases is noted as a limitation in Section 5.4. Because these cases account for 28.7% of the analytic sample, a sensitivity analysis reestimating the structural model on the 124 main‐study respondents alone is reported in Section 4.3.6.

### 3.3. Instrument Design and Constructs

A five‐point Likert‐scale questionnaire (1 = Strongly Disagree to 5 = Strongly Agree) was developed following Churchill [48] and Straub [49]. Items for each construct were adapted from validated scales: perceived ease of use and perceived usefulness from Davis [17] and Tubaishat [20]; digital literacy adoption from Tischendorf et al. [31]; academic performance from McGregory [50] and Roll [51]; and professional preparedness from Qin et al. [52]. The full item wording for all constructs is provided in Appendix A.

Two measurement decisions warrant explicit statement. First, academic performance was measured as students’ self‐reported perceptions of their own academic performance, not through objective records such as GPA, examination scores, or clinical assessments. Objective records were not used because institutional policy does not permit the release of individual examination results, and linking survey responses to academic records would have compromised the anonymity assured to participants. Second, professional preparedness was likewise measured as a self‐rated perception, consistent with the source scale [52], rather than through external clinical evaluations or licensing outcomes. Both decisions mean that the outcome constructs share method variance with the predictor constructs; the procedures used to assess the resulting risk of common method bias are described in Section 3.4, and the interpretive consequences are discussed in Sections 4.5 and 5.4. Table 3 summarizes the constructs and their item composition.

**TABLE 3:** Constructs and number of items.

Construct	Items	Adapted from
Perceived Ease of Use (PEU)	5	Davis [17]; Tubaishat [20]
Perceived Usefulness (PU)	5	Davis [17]; Tubaishat [20]
Adoption of Digital Literacy (ADL)	5	Tischendorf et al. [31]
Academic Performance (AP)	5	McGregory [50]; Roll [51]
Professional Preparedness (PP)	5 (4 retained)	Qin et al. [52]

*Note:* Source: Authors’ construct (2025).

### 3.4. Data Analysis: PLS‐SEM

Data were analyzed using PLS‐SEM in SmartPLS 4 [53], following the two‐step assessment approach: (1) measurement model evaluation (reliability and validity) followed by (2) structural model evaluation [46]. PLS‐SEM was preferred over covariance‐based SEM because it makes no distributional assumptions, is well suited to small and nonnormal datasets, and provides effect size and predictive relevance measures [54, 55]. Bootstrapping with 5000 subsamples and two‐tailed tests at the conventional 5% significance level (critical *t* ≥ 1.96) was used for hypothesis testing [56]. Throughout the manuscript, “effect” is used in its conventional statistical sense to denote a standardized model coefficient or a product of such coefficients. It does not denote a causal effect, and the design supports associational interpretation only.

The specific indirect effect for each mediated pathway implied by the model was obtained directly from the PLS‐SEM bootstrap routine in SmartPLS. Because the sampling distribution of a product of coefficients is generally nonnormal, nonparametric bootstrapping of the indirect effect was used [57, 58]. Percentile 95% confidence intervals were computed from the same 5000 subsamples used for the direct paths; an interval excluding zero indicates a statistically significant indirect effect [59]. Cohen’s *f* ^2^ effect sizes were taken from the SmartPLS *f* ^2^ output, defined as the change in an endogenous construct’s *R* ^2^ when the focal predictor is omitted, and divided by the unexplained variance of that construct [46]. Out‐of‐sample predictive power was assessed with PLSpredict (10 folds, 10 repetitions) and the cross‐validated predictive ability test, CVPAT [60, 61]. Confidence intervals for the heterotrait–monotrait (HTMT) ratios were obtained by percentile bootstrapping with 5000 subsamples [62].

Common method bias was addressed procedurally and statistically. Procedurally, respondents were assured of anonymity and informed there were no right or wrong answers [63]. Statistically, the full collinearity procedure of Kock [64] was applied, as reported in Section 4.3.5. No marker variable was included in the instrument, so marker‐based diagnostics were not available.

### 3.5. Ethical Considerations

Ethical clearance for this study was granted by the Research Ethics Committee of the University for Development Studies on 15 January 2025 (reference UDS/RB/0199/25). The University for Development Studies is the authors’ home institution. Permission to administer the survey on site was granted separately by the management of the Nursing and Midwifery Training College, Tamale, on 20 January 2025. Both approvals were in place before the pilot study and before main data collection, and no data of any kind were collected before the later of the two dates. All participants received a written information sheet describing the study’s purpose, the voluntary nature of participation, and their right to withdraw at any time without consequence. All participants were aged 18 years or older (youngest participant: 18 years), so parental or guardian consent and participant assent were not required. Each participant gave informed consent before data collection: electronically for the Google Forms mode and in writing for the paper mode. No identifying information was collected, and responses were stored on a password‐protected computer accessible only to the research team.

## 4. Results and Discussion

### 4.1. Demographic Profile of Respondents

Table 4 presents the demographic distribution of the 174 respondents. The sample was 55.7% female (97 respondents). The majority of respondents (52.9%) fell within the lowest age category of the questionnaire (15–25 years), reflecting the typical age profile of students in preservice nursing and midwifery programs; the youngest respondent was 18 years old. The 26–35 age group represented 35.6%, indicating a sizable proportion of mature or returning students. Only gender and age were collected; program of enrollment, year of study, prior technology exposure, and access to personal devices were not recorded, which limits the demographic characterization of the sample and is noted as a limitation in Section 5.4.

**TABLE 4:** Demographic distribution of respondents.

Demographic	Category	Frequency	Percentage
Gender	Male	77	44.3
	Female	97	55.7
	Total	174	100.0

Age	15–25 years	92	52.9
	26–35 years	62	35.6
	36–45 years	15	8.6
	46–55 years	5	2.9
	Total	174	100.0

*Note:* Age was recorded in the questionnaire’s categories. The lowest category was 15–25 years; no participant was younger than 18. Source: Authors’ construction (2025).

### 4.2. Measurement Model Assessment

The measurement model was assessed for indicator reliability, internal consistency reliability, convergent validity, and discriminant validity following Hair et al. [46].

#### 4.2.1. Indicator Reliability and the PLS Outer Loading Model

Indicator reliability is assessed by examining outer loadings, which should be at least 0.708 so that each indicator accounts for more than 50% of the variance of its assigned construct [46]. One indicator, PP5, recorded an outer loading of 0.662 in the initial estimation and was removed before the final model estimation, so Professional Preparedness was measured with four indicators (PP1 to PP4). All other constructs retained their full five‐item sets, as shown in Figure 2. In the final model, loadings ranged from 0.725 (PEU1) to 0.943 (PP4). PEU1 has the lowest loading but exceeds the 0.708 threshold and was therefore retained. Every measurement and structural statistic reported in Sections 4.2 and 4.3 was computed from this final specification.

**FIGURE 2:**
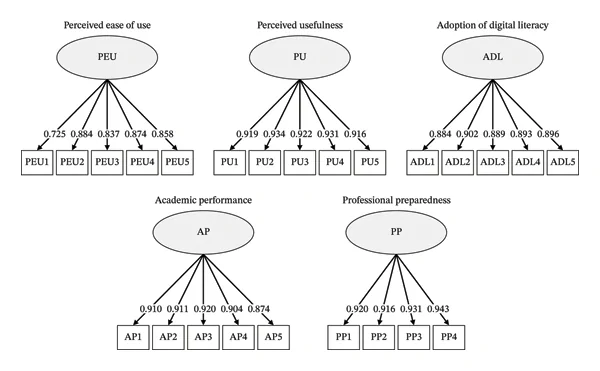
Measurement model with standardized outer loadings. Note: Standardized outer loadings from the final model. All loadings exceed the recommended threshold of 0.708 [46]. PP5 (loading 0.662) was removed before final estimation. PEU = Perceived Ease of Use; PU = Perceived Usefulness; ADL = Adoption of Digital Literacy; AP = Academic Performance; PP = Professional Preparedness. Source: Authors’ construction from SmartPLS output (2025).

#### 4.2.2. Internal Consistency Reliability

Internal consistency was evaluated using Cronbach’s alpha, rho_A, and composite reliability (rho_c). Cronbach’s alpha ranged from 0.892 (PEU) to 0.957 (PU), above the recommended 0.70 threshold [65], and rho_A values, proposed by Dijkstra and Henseler [66], ranged from 0.898 to 0.958. Composite reliability ranged from 0.921 (PEU) to 0.967 (PU). Hair et al. [46] treat values of 0.95 and above as a sign that items may be redundant, and four constructs exceed this value: ADL (0.952), AP (0.957), PP (0.961), and PU (0.967). Several items within these scales are close paraphrases of one another (Appendix A), so part of the high reliability probably reflects item redundancy rather than breadth of measurement; this is noted as a limitation in Section 5.4. Convergent validity was supported by Average Variance Extracted (AVE) values between 0.702 and 0.861, all above the 0.50 threshold [67]. Table 5 summarizes these results.

**TABLE 5:** Construct reliability and convergent validity.

Construct	Cronbach’s alpha	rho_A	Composite reliability (rho_c)	AVE
Adoption of Digital Literacy (ADL)	0.936	0.937	0.952	0.797
Academic Performance (AP)	0.944	0.946	0.957	0.817
Perceived Ease of Use (PEU)	0.892	0.898	0.921	0.702
Professional Preparedness (PP)	0.946	0.946	0.961	0.861
Perceived Usefulness (PU)	0.957	0.958	0.967	0.855
Threshold	≥ 0.70	≥ 0.70	0.70 to 0.95	≥ 0.50

*Note:* Source: SmartPLS output (authors’ construction, 2025).

#### 4.2.3. Discriminant Validity

Discriminant validity was assessed using cross‐loadings, the Fornell–Larcker criterion, and the HTMT ratio. For cross‐loadings (Table 6), each indicator loaded more strongly on its assigned construct than on any other.

**TABLE 6:** Indicator cross‐loadings.

Indicator	ADL	AP	PEU	PP	PU
ADL1	0.884	0.716	0.630	0.586	0.732
ADL2	0.902	0.734	0.667	0.592	0.751
ADL3	0.889	0.712	0.633	0.585	0.730
ADL4	0.893	0.717	0.633	0.582	0.734
ADL5	0.896	0.730	0.609	0.650	0.715
AP1	0.719	0.910	0.645	0.677	0.756
AP2	0.765	0.911	0.675	0.674	0.798
AP3	0.795	0.920	0.691	0.736	0.778
AP4	0.717	0.904	0.664	0.677	0.743
AP5	0.648	0.874	0.623	0.634	0.690
PEU1	0.533	0.516	0.725	0.497	0.535
PEU2	0.596	0.627	0.884	0.552	0.712
PEU3	0.614	0.658	0.837	0.587	0.657
PEU4	0.642	0.641	0.874	0.576	0.696
PEU5	0.588	0.608	0.858	0.558	0.648
PP1	0.573	0.683	0.590	0.920	0.608
PP2	0.616	0.687	0.603	0.916	0.650
PP3	0.668	0.715	0.636	0.931	0.670
PP4	0.631	0.707	0.627	0.943	0.652
PU1	0.771	0.766	0.678	0.619	0.919
PU2	0.752	0.788	0.716	0.653	0.934
PU3	0.772	0.797	0.732	0.668	0.922
PU4	0.766	0.763	0.735	0.639	0.931
PU5	0.731	0.742	0.738	0.636	0.916

*Note:* All indicators load highest on their designated construct. Source: SmartPLS output (authors’ construction, 2025).

The Fornell–Larcker criterion (Table 7) supported discriminant validity, as the square root of each construct’s AVE exceeded its correlations with all other constructs. The HTMT analysis (Table 8) showed all values below the 0.90 threshold for conceptually related constructs [62]. Three ratios exceed the stricter 0.85 threshold: PU‐AP (0.876), PU‐ADL (0.866), and AP‐ADL (0.858). For these three pairs, the upper limit of the bootstrapped 95% confidence interval also exceeds 0.90 (PU‐AP 0.913, PU‐ADL 0.909, AP‐ADL 0.908), although every interval excludes 1.00.

**TABLE 7:** Discriminant validity by the Fornell–Larcker criterion (square roots of AVE on diagonal).

Construct	ADL	AP	PEU	PP	PU
Adoption of Digital Literacy (ADL)	0.893				
Academic Performance (AP)	0.808	0.904			
Perceived Ease of Use (PEU)	0.711	0.731	0.838		
Professional Preparedness (PP)	0.671	0.753	0.662	0.928	
Perceived Usefulness (PU)	0.820	0.834	0.779	0.696	0.924

*Note:* Diagonal values = square root of AVE. Source: SmartPLS output (authors’ construction, 2025).

**TABLE 8:** Discriminant validity by HTMT ratios.

Construct	ADL	AP	PEU	PP	PU
ADL					
AP	0.858				
PEU	0.777	0.794			
PP	0.712	0.795	0.721		
PU	0.866	0.876	0.840	0.731	

*Note:* All HTMT values < 0.90 [62]. Values for PU‐AP (0.876), PU‐ADL (0.866), and AP‐ADL (0.858) exceed the stricter 0.85 benchmark and are discussed in the text; their 95% percentile bootstrap confidence intervals are [0.831, 0.913], [0.814, 0.909], and [0.793, 0.908]. Source: SmartPLS output; confidence intervals from percentile bootstrapping of the study data (authors’ computation, 2025).

These three ratios warrant comment rather than a bare pass/fail judgment. The proximity of PU and ADL is plausible on substantive grounds: for students, believing that digital tools are useful and reporting that one has adopted them may be difficult to separate, particularly where tool use is embedded in coursework rather than freely chosen. Three of the five AP items also refer to digital tools (Appendix A), which ties academic performance closely to both beliefs and adoption. Three observations argue for retaining the constructs as distinct. First, the Fornell–Larcker criterion is satisfied (e.g., √AVE for ADL = 0.893 and PU = 0.924, both exceeding their intercorrelation of 0.820). Second, every bootstrapped HTMT interval excludes 1.00. Third, the constructs derive from different source scales measuring belief (PU), reported behavior (ADL), and perceived outcomes (AP). Nevertheless, the data do not establish discriminant validity against the 0.90 criterion for these pairs, and the consequences extend beyond a single path. The PU ⟶ ADL coefficient (H5, *β* = 0.678) may be upwardly biased because of incomplete construct separation. The PU ⟶ AP and ADL ⟶ AP coefficients (H3 and H6) are estimated in one equation whose predictors overlap substantially with each other and with the outcome, which is consistent with the elevated inner VIF of 4.008 for PU ⟶ AP and means that the division of explained variance among the predictors of AP is not sharply identified. Every indirect effect routed through adoption (PEU ⟶ PU ⟶ ADL, PU ⟶ ADL ⟶ AP, PEU ⟶ PU ⟶ ADL ⟶ AP, and ADL ⟶ AP ⟶ PP) inherits the same ambiguity. H3, H5, and H6, and the adoption‐mediated pathways are therefore the least secure results in the model, and the recommendations drawn from them in Section 5.3 are correspondingly provisional. Future studies in comparable populations should consider behavioral adoption measures (e.g., platform usage logs) and objective performance records to sharpen these distinctions.

### 4.3. Structural Model Assessment

#### 4.3.1. Multicollinearity

Multicollinearity among predictor constructs was assessed using Variance Inflation Factor (VIF) values against the ≤ 5.0 threshold of Hair et al. [45]. As shown in Table 9, all VIF values remained below this threshold. The highest value was 4.008 (PU ⟶ AP), which exceeds the stricter 3.3 benchmark; ADL ⟶ AP (3.187) is close to it. These are inner VIF values: each describes collinearity among the predictors of a single endogenous construct. They are not the full collinearity VIFs of Kock [64], which are obtained by regressing each latent construct on all remaining constructs, and they are not interpreted here as a common method bias diagnostic. The full collinearity assessment is reported separately in Section 4.3.5.

**TABLE 9:** Multicollinearity statistics (inner VIF values).

Predictor	Endogenous construct	VIF	Decision
Adoption of Digital Literacy (ADL)	Academic Performance (AP)	3.187	No multicollinearity
Academic Performance (AP)	Professional Preparedness (PP)	1.000	No multicollinearity
Perceived Ease of Use (PEU)	Adoption of Digital Literacy (ADL)	2.542	No multicollinearity
Perceived Ease of Use (PEU)	Academic Performance (AP)	2.648	No multicollinearity
Perceived Ease of Use (PEU)	Perceived Usefulness (PU)	1.000	No multicollinearity
Perceived Usefulness (PU)	Adoption of Digital Literacy (ADL)	2.542	No multicollinearity
Perceived Usefulness (PU)	Academic Performance (AP)	4.008	Below 5.0; above 3.3

*Note:* Inner (path‐specific) VIF values describing collinearity among the predictors of each endogenous construct. Threshold ≤ 5.0 [45]; stricter benchmark 3.3. These are not full collinearity VIFs; for the full collinearity assessment, see Table 10 in Section 4.3.5. Source: SmartPLS output (authors’ construction, 2025).

**TABLE 10:** Common method bias screening with full collinearity VIF values [64].

Construct	Full collinearity VIF	Result (criterion ≤ 3.3)
Adoption of Digital Literacy (ADL)	3.689	Above 3.3
Academic Performance (AP)	4.589	Above 3.3
Perceived Ease of Use (PEU)	2.820	Below 3.3
Professional Preparedness (PP)	2.491	Below 3.3
Perceived Usefulness (PU)	4.790	Above 3.3

*Note:* Each construct was regressed on all remaining constructs. Kock [64] treats values above 3.3 as an indication that common method bias may be present. Source: Authors’ computation from SmartPLS latent variable scores (2025).

#### 4.3.2. Path Coefficient Significance and Hypothesis Testing

Path coefficients were assessed using bootstrapping with 5000 subsamples at the conventional two‐tailed 5% significance level (critical *t* ≥ 1.96). All seven hypothesized paths were significant at this level in the full sample, and every percentile 95% confidence interval excludes zero. The two weakest paths are PEU ⟶ AP (H1, *β* = 0.142, *t* = 2.477, *p* = 0.013) and PEU ⟶ ADL (H4, *β* = 0.183, *t* = 2.603, *p* = 0.009); their robustness to exclusion of the pilot respondents is examined in Section 4.3.6. Figure 3 shows the structural model with standardized coefficients, *t*‐statistics, and *R* ^2^ values, and Table 11 reports the detailed results. The measurement model is shown separately in Figure 2.

**FIGURE 3:**
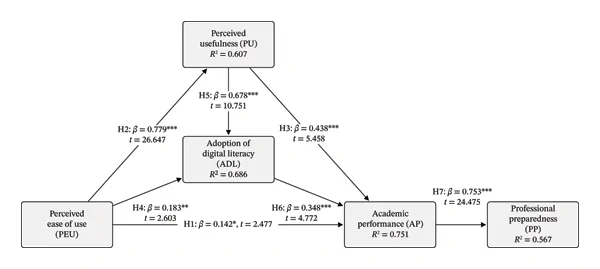
Structural model with hypothesis testing results. Note: *β* = standardized path coefficient; *t* = *t*‐statistic from bootstrapping (5000 subsamples). Significance: ^∗∗∗^ *p* < 0.001, ^∗∗^ *p* < 0.01, and ^∗^ *p* < 0.05 (two‐tailed, critical *t* ≥ 1.96). *R* ^2^ values are shown within the construct boxes. Indicator loadings are reported in Figure 2. PEU = Perceived Ease of Use; PU = Perceived Usefulness; ADL = Adoption of Digital Literacy; AP = Academic Performance; PP = Professional Preparedness. Source: Authors’ construction from SmartPLS output (2025).

**TABLE 11:** Hypothesis testing results for direct relationships.

Hyp.	Relationship	*β*	STDEV	*t*	*p*	95% CI	Decision
H1	PEU ⟶ AP	0.142^∗^	0.057	2.477	0.013	[0.027, 0.252]	Supported
H2	PEU ⟶ PU	0.779^∗∗∗^	0.029	26.647	< 0.001	[0.717, 0.831]	Supported
H3	PU ⟶ AP	0.438^∗∗∗^	0.080	5.458	< 0.001	[0.283, 0.595]	Supported
H4	PEU ⟶ ADL	0.183^∗∗^	0.070	2.603	0.009	[0.048, 0.325]	Supported
H5	PU ⟶ ADL	0.678^∗∗∗^	0.063	10.751	< 0.001	[0.546, 0.798]	Supported
H6	ADL ⟶ AP	0.348^∗∗∗^	0.073	4.772	< 0.001	[0.205, 0.495]	Supported
H7	AP ⟶ PP	0.753^∗∗∗^	0.031	24.475	< 0.001	[0.688, 0.810]	Supported

*Note: β* = standardized path coefficient; STDEV = bootstrap standard deviation; 95% CI = percentile bootstrap confidence interval (5000 subsamples); critical *t* ≥ 1.96 at the 5% significance level (two‐tailed). All results refer to the full sample (*n* = 174); see Table 12 for *n* = 124. Source: SmartPLS output (authors’ construction, 2025).

^∗^ *p* < 0.05.

^∗∗^ *p* < 0.01.

^∗∗∗^ *p* < 0.001.

**TABLE 12:** Effect sizes (*f* ^2^) for structural paths.

Path	*f* ^2^	Interpretation
PEU ⟶ AP	0.031	Small
PEU ⟶ PU	1.542	Large
PU ⟶ AP	0.191	Medium
PEU ⟶ ADL	0.042	Small
PU ⟶ ADL	0.577	Large
ADL ⟶ AP	0.153	Medium
AP ⟶ PP	1.308	Large

*Note:* *f* ^2^ = (*R* ^2^ included − *R* ^2^ excluded)/(1 − *R* ^2^ included), where *R* ^2^ excluded is the *R* ^2^ of the endogenous construct with the focal predictor omitted [46]. The *R* ^2^ values used are listed in Appendix B. Source: SmartPLS output (authors’ construction, 2025).

#### 4.3.3. Model Explanatory Power (*R* ^2^) and Effect Sizes (*f* ^2^)

Table 13 reports *R* ^2^ values. The model explains 75.1% of the variance in self‐rated academic performance (*R* ^2^ = 0.751) and 68.6% in adoption, both classified as substantial under Chin [68], and 60.7% in perceived usefulness and 56.7% in professional preparedness, both classified as moderate. Two qualifications apply. First, because all constructs share a single self‐report method, part of this explained variance may reflect common method variance rather than substantive association; Section 4.3.5 reports the relevant diagnostics. Second, the *R* ^2^ of 0.607 for perceived usefulness derives from a single predictor (PEU, *β* = 0.779). A single belief construct accounting for over 60% of the variance in another belief construct indicates very tight coupling between PEU and PU in this population. The inner VIF does not flag collinearity on this path (VIF = 1.000, as PU has one predictor), but the PU‐PEU HTMT ratio (0.840) is close to the 0.85 benchmark, and respondents may not sharply distinguish finding tools easy from finding them useful. This does not invalidate the TAM structure, which predicts a strong PEU ⟶ PU association, but it cautions against reading the two constructs as fully independent sources of information in this sample.

**TABLE 13:** Explanatory power (*R* ^2^) and predictive relevance (*Q* ^2^ predict).

Endogenous construct	*R* ^2^	*R* ^2^ adjusted	Classification [68]	*Q* ^2^ predict
Academic Performance (AP)	0.751	0.746	Substantial (> 0.67)	0.530
Adoption of Digital Literacy (ADL)	0.686	0.683	Substantial (> 0.67)	0.499
Professional Preparedness (PP)	0.567	0.564	Moderate (> 0.33)	0.423
Perceived Usefulness (PU)	0.607	0.604	Moderate (> 0.33)	0.603

*Note:* Source: SmartPLS output (authors’ construction, 2025).

Out‐of‐sample predictive power was assessed with PLSpredict [60]. All Q^2^predict values exceed zero, at the construct level (Table 13, 0.423–0.603) and at the indicator level (0.337–0.540), so the model predicts better than the naive benchmark of indicator means. For each of the 19 indicators of the endogenous constructs, the PLS‐SEM prediction error (RMSE) is lower than that of the linear regression benchmark, which Shmueli et al. [60] classify as high predictive power. The cross‐validated predictive ability test [61] shows that the model predicts better than the indicator‐average benchmark for all four endogenous constructs (*p* < 0.001) and has a lower overall loss than the linear model benchmark (average loss difference = −0.025, *p* = 0.006).

Cohen’s *f* ^2^ effect sizes for each structural path are reported in Table 12, interpreted against the conventional benchmarks of 0.02 (small), 0.15 (medium), and 0.35 (large) [46]. The values are the SmartPLS *f* ^2^ statistics: the change in *R* ^2^ of the endogenous construct when the focal predictor is omitted, divided by the unexplained variance of that construct. The *R* ^2^ values with and without each predictor are reported in Appendix B. In the two single‐predictor equations (PEU ⟶ PU and AP ⟶ PP), *f* ^2^ reduces to *R* ^2^/(1 − *R* ^2^). Explanatory power is concentrated in these two paths and in PU ⟶ ADL (*f* ^2^ = 0.577). PU ⟶ AP and ADL ⟶ AP have medium effects, while the effects of perceived ease of use on adoption (*f* ^2^ = 0.042) and on academic performance (*f* ^2^ = 0.031) are small, although both reach statistical significance in the full sample.

#### 4.3.4. Mediation Analysis: Indirect and Total Effects

Because the conceptual model frames a sequential pathway (PEU/PU ⟶ adoption ⟶ academic performance ⟶ professional preparedness), the direct paths in Table 11 describe the structural model only partially. Table 2 reports the specific indirect effects for the mediated pathways, taken directly from the SmartPLS bootstrap output (5000 subsamples; see Section 3.4). An indirect effect is treated as significant where its percentile 95% confidence interval excludes zero [59].

The pattern in Table 2 is consistent with partial rather than full mediation of the PEU‐AP association, on the ordering the model specifies. Of the total PEU ⟶ AP association of 0.731, about four‐fifths (0.588) is carried by indirect pathways: through perceived usefulness (0.341), through the PU ⟶ ADL chain (0.184), and through adoption alone (0.064). The last of these is small, but its confidence interval (0.016–0.123) excludes zero. The direct path (0.142) is significant in the full sample but, as Section 4.3.6 shows, not when the pilot respondents are excluded, so the evidence for a direct association beyond the mediated routes is weak. The ADL ⟶ AP ⟶ PP indirect effect (0.262) is consistent with the sequential ordering the model specifies, from adoption through academic performance to professional preparedness, although the cross‐sectional design cannot establish that ordering and the adoption‐mediated pathways carry the discriminant validity caveat set out in Section 4.2.3.

#### 4.3.5. Common Method Bias Assessment

Because every construct was measured through self‐report in a single questionnaire at one time point, common method bias is a live threat to the validity of the estimates [63]. Following Kock [64], each of the five latent constructs was regressed in turn on the four remaining constructs, using the latent variable scores of the final model, and the resulting full collinearity VIF was compared against the 3.3 criterion. Table 10 reports the results. These values are distinct from the inner VIF values in Table 9, which describe collinearity among the predictors of individual endogenous constructs.

Three of the five full collinearity VIFs exceed the 3.3 criterion: PU (4.790), AP (4.589), and ADL (3.689). By Kock [64] criterion, common method bias cannot be ruled out for these constructs. The procedure cannot separate method variance from genuine overlap between constructs, and the three constructs with high values are the same three whose HTMT ratios exceed 0.85 (Section 4.2.3), so part of the elevation may reflect construct proximity rather than method effects. Either explanation weakens confidence in the estimates. No marker variable was included in the survey design, so marker‐based diagnostics could not be applied (Section 5.4). Several features of the study leave the estimates exposed to method variance. Every construct was self‐reported, all constructs were measured in the same questionnaire at one time point, academic performance and preparedness are self‐perceptions rather than objective outcomes, and the *R* ^2^ for self‐rated academic performance is high (0.751). The statistical significance of all seven hypothesized paths in the full sample, several with coefficients above 0.65, is consistent both with TAM’s predictions and with method‐variance inflation, and the two explanations cannot be separated within this design. The coefficients discussed in Sections 4.4 and 5 should therefore be read as estimates that may be inflated by the shared method variance.

#### 4.3.6. Sensitivity Analysis: Estimates Excluding the Pilot Respondents

The 50 pilot respondents (records R001 to R050 in the dataset) account for 28.7% of the analytic sample. Retaining them is defensible on the grounds set out in Section 3.2: the instrument was unchanged after the pilot, the pilot participants were drawn from the same population, and each responded once, but a share that large means the substantive conclusions should not depend on their inclusion. The structural model was therefore reestimated on the 124 main‐study respondents alone (records R051 to R174), using an identical specification: the same indicators, the same seven direct paths, the same PLS‐SEM algorithm settings, and the same bootstrap settings (5000 subsamples, percentile confidence intervals, two‐tailed, 5% level). For each of H1 to H7, Table 14 reports the coefficient in the full sample, the coefficient in the *n* = 124 sample, the difference between them (Δβ), and the bootstrap 95% confidence interval and *p* value in the *n* = 124 sample.

**TABLE 14:** Sensitivity analysis of direct path coefficients with and without the pilot respondents.

Hyp.	Path	*β* (*n* = 174)	*β* (*n* = 124)	Δ*β*	95% CI (*n* = 124)	*p* (*n* = 124)
H1	PEU ⟶ AP	0.142	0.085	−0.057	[−0.068, 0.231]	0.266
H2	PEU ⟶ PU	0.779	0.787	0.008	[0.717, 0.846]	< 0.001
H3	PU ⟶ AP	0.438	0.423	−0.015	[0.211, 0.634]	< 0.001
H4	PEU ⟶ ADL	0.183	0.211	0.028	[0.044, 0.375]	0.013
H5	PU ⟶ ADL	0.678	0.646	−0.032	[0.482, 0.794]	< 0.001
H6	ADL ⟶ AP	0.348	0.399	0.051	[0.226, 0.578]	< 0.001
H7	AP ⟶ PP	0.753	0.786	0.033	[0.722, 0.842]	< 0.001

*Note:* Δ*β* = *β* (*n* = 124) minus *β* (*n* = 174), computed from the rounded coefficients. The 95% confidence interval and *p* value refer to the *n* = 124 model (5000 bootstrap subsamples, percentile intervals, two‐tailed). Both models use the same indicators and identical algorithm settings. Source: Authors’ computation from the study data (2025).

Two points bear on how Table 14 should be read. First, the reduced sample of 124 still exceeds the a priori power requirement of 119 identified in Section 3.2, but the margin is small, so wider confidence intervals are expected on statistical grounds alone. Second, the pilot and main‐study respondents were drawn from the same institution and the same programs, so this analysis tests robustness to the timing and framing of data collection, not to differences in the population.

Six of the seven paths remained statistically significant in the *n* = 124 sample, and no coefficient changed sign. The exception was the direct association between perceived ease of use and academic performance (H1), which was significant in the full sample (*β* = 0.142, *p* = 0.013) but not without the pilot respondents (*β* = 0.085, *p* = 0.266, 95% CI [‐0.068, 0.231]). H1 is therefore not robust to exclusion of the pilot respondents and is interpreted accordingly in Sections 4.4.1 and 5.1. The association between perceived ease of use and adoption (H4), the second‐weakest path in the full sample, remained significant (*β* = 0.211, *p* = 0.013, 95% CI [0.044, 0.375]). The largest change in any coefficient was Δβ = −0.057 on H1; all other changes were 0.051 or smaller in absolute value.

### 4.4. Discussion of Findings

#### 4.4.1. H1: Perceived Ease of Use and Academic Performance

The positive association between perceived ease of use and self‐rated academic performance in the full sample (*β* = 0.142, *t* = 2.477, *p* = 0.013) supports H1 and is consistent with the cognitive‐load rationale set out in Section 2.5.1: when tools are easy to navigate, attention is available for learning content rather than technology management [17, 36]. Two results qualify this. First, the association was not robust to the exclusion of the pilot respondents (Table 14), so H1 receives only fragile support and should not be treated as an established finding. Second, most of the PEU‐AP association runs through perceived usefulness and adoption (Table 2): the direct path accounts for about one‐fifth of the total effect and has a small effect size (*f* ^2^ = 0.031). This pattern is closer to TAM’s original formulation, in which ease of use operates mainly through usefulness and use, than to the direct mechanism proposed in Section 2.5.1. In the context of the Nursing and Midwifery Training College, Tamale, where students may have limited prior technology experience, intuitive tool design may still matter for effective digital engagement, though the cross‐sectional design cannot establish that ease of use produces academic gains.

#### 4.4.2. H2: Perceived Ease of Use and Perceived Usefulness

The strong association between perceived ease of use and perceived usefulness (*β* = 0.779, *t* = 26.647, *p* < 0.001), the strongest in the model, supports H2 and is consistent with TAM’s proposition that ease of use is a prerequisite for perceptions of utility [34]. The finding matches Holden and Karsh [19] in healthcare technology contexts. As noted in Section 4.3.3, the sheer strength of this association also suggests that the two belief constructs are tightly coupled in this population, which counsels some caution about treating them as fully independent.

#### 4.4.3. H3: Perceived Usefulness and Academic Performance

The positive association between perceived usefulness and self‐rated academic performance (*β* = 0.438, *t* = 5.458, *p* < 0.001) supports H3 [17]. For these students, this is consistent with the practical benefit of digital resources (simulation platforms, clinical databases, and e‐learning tools) for engagement with nursing theory and clinical practice [2]. The larger coefficient relative to perceived ease of use (0.438 vs. 0.142) suggests that utility perceptions are more strongly tied to self‐assessed outcomes than usability perceptions, consistent with the broader TAM literature [38]. Because the PU‐AP HTMT ratio is 0.876 and PU ⟶ AP has the highest inner VIF (4.008), the size of this coefficient should be read with the caution set out in Section 4.2.3.

#### 4.4.4. H4: Perceived Ease of Use and Adoption of Digital Literacy

Perceived ease of use shows a small positive association with adoption (*β* = 0.183, *t* = 2.603, *p* = 0.009), supporting H4, and the association remained significant when the pilot respondents were excluded (*β* = 0.211, *p* = 0.013; Table 14). Its effect size is small (*f* ^2^ = 0.042, Table 12), so usability accounts for little variance in adoption beyond what usefulness already accounts for. For these students, adoption is tied far more to perceived clinical and academic utility than to usability. If this pattern reflects a substantive relationship, it would favor demonstrating the practical value of digital tools over interface simplification in technology integration strategies [13], though an association measured at a single time point cannot establish that ordering.

#### 4.4.5. H5: Perceived Usefulness and Adoption of Digital Literacy

Perceived usefulness shows a strong association with digital literacy adoption (*β* = 0.678, *t* = 10.751, *p* < 0.001), supporting H5 and consistent with Legris et al. [38] on usefulness as the dominant adoption correlate. The much larger coefficient compared to perceived ease of use (0.678 vs. 0.183) indicates that these students’ reported adoption tracks perceived academic and clinical benefit. This is among the least secure estimates in the model. As set out in Section 4.2.3, the PU‐ADL HTMT ratio of 0.866 exceeds the stricter 0.85 criterion, its confidence interval reaches 0.909, and the two constructs are conceptually close, so part of the coefficient may reflect imperfect separation between a belief and a self‐report of the behavior that belief is expected to predict, rather than a relationship between distinct constructs. The shared‐method concern in Section 4.3.5 works in the same direction, and the coefficient may be upwardly biased because of incomplete construct separation. The result remains compatible with strategies such as peer testimonials, faculty endorsements, and clinical application demonstrations for building adoption, but neither the magnitude of the coefficient nor the indirect effects that pass through adoption should be relied on as precise estimates.

#### 4.4.6. H6: Adoption of Digital Literacy and Academic Performance

The positive association between digital literacy adoption and self‐rated academic performance (*β* = 0.348, *t* = 4.772, *p* < 0.001) supports H6. Students who report actively adopting digital tools also report stronger academic performance, consistent with access to broader information resources, collaborative learning, and critical‐thinking development [37, 39]. Because performance was self‐rated, this association should be read as students’ adoption tracking their own assessment of academic success, not as demonstrated gains in measured achievement. This path is also estimated in the same equation as perceived usefulness, where the inner VIF of 3.187 and the overlap among PU, ADL, and AP discussed in Section 4.2.3 mean the division of explained variance between adoption and usefulness is not sharply identified.

#### 4.4.7. H7: Academic Performance and Professional Preparedness

The strong association between self‐rated academic performance and professional preparedness (*β* = 0.753, *t* = 24.475, *p* < 0.001) supports H7 and is the second strongest relationship in the model. It is consistent with the expectation that nursing academic achievement and professional readiness go together [29, 40]. Since both constructs are self‐perceptions measured in the same instrument, part of this association may reflect a general self‐evaluative disposition; students confident in their academic standing may also rate themselves as better prepared. Objective performance records and external preparedness assessments would be needed to separate substantive association from shared self‐perception.

### 4.5. Integration and Interpretive Boundaries

Taken together, the findings are consistent with a TAM‐based chain: perceived ease of use is associated with perceived usefulness (H2) and adoption (H4); perceived usefulness with adoption (H5) and performance (H3); adoption with performance (H6); and academic performance with professional preparedness (H7). The direct association between ease of use and performance (H1) is the exception: it is small and did not survive the exclusion of the pilot respondents. The bootstrapped indirect effects in Table 2 quantify the sequential structure of this chain rather than leaving it as narrative, but they describe the ordering the model assumes rather than establishing it.

Four interpretive boundaries apply. First, the full‐sample results (seven of seven hypothesized paths significant, several coefficients above 0.65, and *R* ^2^ up to 0.751) should not be read solely as confirmation of TAM’s applicability. Results this clean are also what common method variance produces when all constructs are self‐reported in one instrument at one sitting [63], and three of the five full collinearity VIFs in Section 4.3.5 exceed Kock’s 3.3 criterion. Second, the cross‐sectional design permits associational claims only. Reverse orderings are plausible for several paths: students performing well academically may come to view digital tools as more useful, rather than the reverse. Third, the single‐site design means the estimates are conditioned on this institution’s characteristics: a rural setting, constrained infrastructure, and a curriculum in which digital tool use may be partly mandated. Each of these could moderate the observed associations: where tool use is compulsory, usability may matter less for adoption than it would under free choice, which may partly explain the small PEU ⟶ ADL coefficient; where devices and connectivity are scarce, perceived usefulness may be shaped as much by access as by belief. Multisite designs sampling institutions that vary on these characteristics are needed to establish the boundary conditions of the model. Fourth, the measurement model limits the claims involving PU, ADL, and AP. Their HTMT ratios (0.858–0.876) sit above the stricter 0.85 criterion, so H3, H5, H6, and every indirect pathway routed through adoption rest on a construct separation the data support only marginally. Where the discussion above refers to what adoption is associated with, that claim is weaker than the corresponding claims about perceived ease of use and professional preparedness.

## 5. Conclusions, Implications, and Recommendations

### 5.1. Summary of Findings

This study examined the associations between digital literacy adoption, self‐rated academic performance, and professional preparedness among nursing and midwifery students at the Nursing and Midwifery Training College, Tamale, Ghana. Using PLS‐SEM with data from 174 students and TAM as the theoretical framework, all seven hypothesized associations reached statistical significance in the full sample, although the direct association between perceived ease of use and academic performance (H1) was not robust to exclusion of the 50 pilot respondents; the other six hypothesized associations did?. Perceived usefulness was the dominant correlate of digital literacy adoption, adoption was positively associated with self‐rated academic performance, and academic performance was strongly associated with professional preparedness. The model explains 75.1% of the variance in academic performance and 56.7% in professional preparedness and shows high out‐of‐sample predictive power, subject to the common‐method caveat in Section 4.3.5.

The three research objectives were addressed as follows. First, perceived usefulness (*β* = 0.678) was far more strongly associated with digital literacy adoption than perceived ease of use (*β* = 0.183, with a small effect size), subject to the construct‐separation caveat set out in Section 4.2.3. Second, digital literacy adoption showed a significant positive association with self‐rated academic performance (*β* = 0.348). Third, academic performance was strongly associated with professional preparedness (*β* = 0.753), and the mediation results indicate that adoption’s association with preparedness runs through academic performance (indirect effect 0.262).

### 5.2. Theoretical Contributions

This study extends TAM to nursing education in a resource‐constrained, rural Ghanaian setting, a context in which few TAM applications have been reported. It documents a full associational pathway from TAM antecedents through adoption to academic and professional outcomes, estimated with bootstrapped indirect effects rather than direct paths alone. The study also responds to calls for contextual TAM applications in developing‐country healthcare education settings [7, 31]. The near‐threshold discriminant validity among PU, ADL, and AP contributes a methodological observation: in populations where tool use is partly mandated, belief and adoption constructs may converge, and future TAM applications in such settings should consider behavioral adoption measures.

### 5.3. Practical and Policy Implications

The implications below rest on cross‐sectional, self‐reported associations from a single institution. They fall into two groups: implications consistent with the observed associations and interventions whose effectiveness requires prospective evaluation.

#### 5.3.1. Implications Consistent With the Observed Associations

Students’ reported adoption tracked perceived usefulness far more closely than ease of use. Educators who introduce digital tools may therefore find it worthwhile to show students how each tool supports specific coursework and clinical tasks, rather than relying on simplified interfaces alone. The strong association between self‐rated academic performance and self‐rated preparedness is consistent with treating digital literacy as part of the academic preparation that students connect with readiness for practice, and with including it among the competencies examined in curriculum reviews.

#### 5.3.2. Interventions Whose Effectiveness Requires Prospective Evaluation

Several measures are plausible but untested by this study: faculty development programs in digital pedagogy, usability testing of institutional platforms, peer mentoring schemes that pair digitally confident students with less confident peers, investment in connectivity and computing devices in rural nursing and midwifery colleges, and standardized digital literacy assessment linked to professional certification. This study did not evaluate any educational intervention, infrastructure investment, or certification policy, and it did not measure objective academic or clinical competence. Each measure would need prospective, preferably controlled, evaluation against objective outcomes before institutions or regulators adopt it on the strength of associations of the kind reported here.

### 5.4. Limitations and Future Research

This study has several limitations, and they bear directly on how the findings should be read.

Measurement. All constructs, including academic performance and professional preparedness, were measured through self‐report in a single questionnaire at one time point. The design is therefore exposed to common method bias: three of the five full collinearity VIFs reported in Section 4.3.5 exceed Kock’s 3.3 criterion, and no marker variable was available for dedicated common‐method diagnostics. Academic performance in particular reflects students’ perceptions of their performance, not GPA, examination scores, or clinical assessments; the high explained variance for this construct (75.1%) may partly reflect shared method variance and shared self‐evaluative disposition rather than substantive association. Several academic performance and preparedness items refer explicitly to digital tools (Appendix A), which build part of the hypothesized relationship into the outcome measures and may inflate their associations with perceived usefulness and adoption. Discriminant validity among perceived usefulness, adoption, and academic performance is also marginal (HTMT 0.858 to 0.876 against the stricter 0.85 criterion), and composite reliability above 0.95 for four constructs suggests some item redundancy; both limit how far H3, H5, H6, and the adoption‐mediated indirect effects can be interpreted.

Design. The cross‐sectional design precludes causal inference. Although TAM specifies directional relationships, the data cannot distinguish these from reverse or reciprocal orderings.

Sampling. Convenience sampling from a single institution limits generalizability, and total enrollment data were not available to express the sample as a proportion of the student population. The 50 pilot respondents were retained in the analytic sample; this is defensible given that no instrument changes followed the pilot and each participant responded once, but it merges pilot and main‐study data collection. The sensitivity analysis in Section 4.3.6 reestimates the model without these cases: six of the seven associations were robust to their exclusion, but the direct association between the perceived ease of use and academic performance (H1) was not. Responses were also collected in two modes (online and paper) without a formal test of mode equivalence. Program of enrollment was not recorded, so the analysis cannot test whether the associations differ between nursing and midwifery students. Several items refer to nursing education or nursing practice, and midwifery respondents may have read them as referring to their own program.

Scope. Only gender and age were recorded, and age only in categories. Program of enrollment, year of study, prior technology exposure, and access to personal devices, all plausibly related to digital literacy in a resource‐constrained setting, were not measured, so their roles as covariates or moderators could not be examined.

Future research should pursue longitudinal designs linking adoption to objective academic records and clinical performance over time; multiinstitutional and cross‐regional studies to establish the boundary conditions identified in Section 4.5; models incorporating social influence (UTAUT), infrastructure quality, and gender‐based digital divide dimensions; behavioral adoption measures such as platform usage logs; and studies of emerging technologies (artificial intelligence, virtual reality) in nursing education.

## Author Contributions

Alhassan Shaibu: conceptualization, methodology, formal analysis, and writing original draft. Iddi Anass: data collection, writing review, and editing.

## Funding

This research received no specific grant from any funding agency in the public, commercial, or not‐for‐profit sectors.

## Disclosure

All the authors read and approved the final manuscript.

## Ethics Statement

The authors declare that this study was conducted in accordance with applicable ethical standards. Ethical clearance was granted by the Research Ethics Committee of the University for Development Studies on 15 January 2025 (reference UDS/RB/0199/25), before the pilot study and the main data collection began. No research data were collected before ethical approval. Separate permission to administer the survey at the Nursing and Midwifery Training College, Tamale, was granted by the College management on 20 January 2025. All participants were aged 18 years or older and gave written or electronic informed consent.

## Conflicts of Interest

The authors declare no conflicts of interest.

## Data Availability

The anonymized dataset, the SmartPLS 4 project file, and the SmartPLS output reports for the full‐sample and *n* = 124 models supporting the findings of this study are available from the corresponding author upon reasonable request. Access to the materials may be provided for purposes of verification and replication of the analyses reported in the article, subject to applicable ethical and confidentiality requirements.

## Appendix A Measurement Items

All items were administered on a five‐point Likert scale (1 = Strongly Disagree, 5 = Strongly Agree), and the wording below is reproduced exactly as administered. Item sources are listed in Table 3. Loadings are standardized outer loadings from the final model. PP5 was removed during measurement‐model assessment because its loading in the initial estimation (0.662) was below the 0.708 threshold; it is shown for completeness. In the dataset, the PEU, PU, and ADL items are coded PEUDT, PUDT, and ADLT. (see Table A1).

**Table A1:** Measurement items, descriptive statistics, and factor loadings.

Code	Item wording (as administered)	Mean (SD)	Loading
PEU1	Digital tools I use in my studies are easy to understand.	3.443 (1.182)	0.725
PEU2	Learning to use digital tools was straightforward for me.	3.506 (1.231)	0.884
PEU3	I find it easy to navigate through the digital platforms provided by the institution.	3.437 (1.181)	0.837
PEU4	I do not need a lot of assistance to use the digital literacy tools effectively.	3.483 (1.212)	0.874
PEU5	Using the digital tools does not require much mental effort.	3.483 (1.168)	0.858
PU1	Digital tools help me accomplish my study tasks more quickly.	3.431 (1.200)	0.919
PU2	Using digital tools improves my academic performance.	3.437 (1.191)	0.934
PU3	The use of digital literacy tools enhances the quality of my studies.	3.431 (1.261)	0.922
PU4	Digital tools make it easier for me to access learning materials.	3.414 (1.204)	0.931
PU5	Overall, I find digital literacy tools to be useful for my academic work.	3.437 (1.215)	0.916
ADL1	I regularly use digital tools to assist with my academic work.	3.477 (1.216)	0.884
ADL2	I incorporate digital literacy tools in my day‐to‐day study activities.	3.437 (1.201)	0.902
ADL3	I am willing to adopt new digital tools that could enhance my studies.	3.471 (1.202)	0.889
ADL4	I frequently use digital resources such as online databases, e‐books, or learning platforms.	3.448 (1.206)	0.893
ADL5	I believe that adopting digital tools is essential for my academic success.	3.443 (1.196)	0.896
AP1	I am satisfied with the grades I have received in my academic courses.	3.454 (1.225)	0.910
AP2	Digital tools have contributed to my improved academic performance.	3.477 (1.178)	0.911
AP3	I believe that my study habits, supported by digital tools, have positively impacted my grades.	3.489 (1.207)	0.920
AP4	I consistently perform well in exams and assignments that utilize digital resources.	3.477 (1.230)	0.904
AP5	My overall academic performance has improved due to the adoption of digital tools.	3.477 (1.221)	0.874
PP1	I feel confident that my academic experience has prepared me for my professional career.	3.431 (1.156)	0.920
PP2	I believe the digital literacy tools I use have enhanced my readiness for professional nursing practice.	3.471 (1.216)	0.916
PP3	I am well‐prepared to apply my academic knowledge to real‐world healthcare settings.	3.506 (1.183)	0.931
PP4	The use of digital tools in my education has equipped me with the skills necessary for professional practice.	3.471 (1.197)	0.943
PP5	I am confident in my ability to use digital tools in a professional healthcare environment.	3.448 (1.206)	0.662 (removed)

## Appendix B *R* ^2^ values underlying the *f* ^2^ effect sizes (Table 12)

For each structural path, the table lists the *R* ^2^ of the endogenous construct in the full model, the *R* ^2^ with the focal predictor omitted, and the resulting *f* ^2^ = (*R* ^2^ included − *R* ^2^ excluded)/(1 − *R* ^2^ included). All values are computed from the latent variable scores of the final SmartPLS model (*n* = 174) and reproduce the SmartPLS *f* ^2^ output. *R* ^2^ values are shown to six decimals so that each *f* ^2^ can be recomputed exactly from the table (see Table A2).

**Table A2:** Effect size (*f*_2_) analysis for the structural model relationships.

Path	Endogenous construct	*R* ^2^ included	*R* ^2^ excluded	*f* ^2^
PEU ⟶ AP	AP	0.750511	0.742871	0.031
PEU ⟶ PU	PU	0.606533	0.000000	1.542
PU ⟶ AP	AP	0.750511	0.702744	0.191
PEU ⟶ ADL	ADL	0.686206	0.673094	0.042
PU ⟶ ADL	ADL	0.686206	0.505199	0.577
ADL ⟶ AP	AP	0.750511	0.712424	0.153
AP ⟶ PP	PP	0.566740	0.000000	1.308

*Note:* For the single‐predictor equations (PU and PP), omitting the predictor leaves *R* ^2^ = 0, so *f* ^2^ = *R* ^2^/(1 − *R* ^2^).
